# Satellite Faculty in an Academic Ophthalmology Department: Junior, Clinical, and Female

**DOI:** 10.1089/whr.2022.0071

**Published:** 2023-05-10

**Authors:** Laxmi Devisetty, Shelby Smith, Irene C. Kuo

**Affiliations:** ^1^ Wilmer Eye Institute, Department of Ophthalmology, University of Michigan, Ann Arbor, Michigan, USA.; ^2^ Department of Ophthalmology, Johns Hopkins University School of Medicine, Baltimore, Maryland, USA.

**Keywords:** academic advancement, academic ophthalmology, faculty satisfaction, leadership, satellite practices, women's status

## Abstract

**Purpose:**

To evaluate the perception of physicians at satellite offices of a large academic ophthalmology department.

**Methods:**

A survey was sent to the 32 physician faculty members working at the satellite offices in the Ophthalmology Department of the University of Michigan. The ophthalmologists answered 44 survey questions on staffing, wait times, physician satisfaction, patient satisfaction, compensation, administrative help, research, and operations management.

**Results:**

Seventeen (53%) satellite ophthalmologists responded. The majority were satisfied with work at satellites, which they felt operated efficiently and believed to feature high patient satisfaction. A minority of ophthalmologists had concerns about salary, volume, marketing support, and geographic location. Some respondents did not understand the compensation structure, satellites' finances, or contribution to the overall department. Most described a lack of research and resident teaching opportunities at satellites.

**Conclusions:**

The perceptions of ophthalmologists who work in satellite offices are important because of the growth of these offices in academic medical centers and the ability for satellite doctors to offer care comparable with and sooner than doctors at the main hospital at locations convenient for patients. Satellite ophthalmologists at this academic center would appreciate increased transparency of compensation and financial structures; administrative help with marketing and maintaining efficiency, which doctors and patients enjoy at satellite offices; and more teaching and research opportunities, which are the basis of academic advancement. Such efforts may help retain satellite doctors, who tend to be junior in rank, female, nontenured faculty, and who experience a higher turnover rate than faculty at the main campus.

## Introduction

Academic medical centers are evolving in response to the challenges of a shifting health care landscape. Just as private practice counterparts established multiple offices to increase access to patients, academic departments have done the same with satellite offices outside the main campus.^1,2^ A major advantage of community-based satellite locations is that patients no longer have to travel long distances to the main hospital of academic institutions or to Veterans Administration medical centers, which tend to be in dense, urban centers. These community locations increase access to health care for patients with sickle cell disease,^3^ patients with mental health issues,^4^ or pediatric populations.^5^ These satellite offices not only have comparable medical outcomes^6^ but also may be preferentially attended over their flagship academic center.^3^

At larger academic ophthalmology departments, satellite offices accounted for more than 50% of total visits and an even higher percentage of department revenue and new patient visits.^7^ Ophthalmology satellite practices serve an important role in the screening, diagnosis, and treatment of diseases such as diabetic retinopathy, the major cause of blindness in working aged Americans.^8^ Unlike private practice, however, academic departments must meet the mandates of their tripartite mission—education and research in addition to patient care.^2,9^ Financial solvency, which traditionally has not been paramount for departments and divisions at the main campus, appears to be the most important factor in the fate of an ophthalmology satellite office.^7^ Therefore, physicians at satellite practices must balance patient care, education, research, and financial success of their satellite.

Examination of how satellite physicians perceive and balance these priorities may help determine the best environment in which they can deliver excellent clinical care, produce scholarly material, and educate trainees while maintaining high job satisfaction, which is important for faculty retention. Clinically focused faculty may lack time for scholarly endeavors, which can lead to lower job satisfaction and disadvantage their career advancement.^10^ Studies also suggest clinical faculty are less likely to be satisfied with their academic promotion, less likely to be in higher academic ranks, and more likely to leave their academic institution.^10–13^

Recent publications surveying ophthalmology chairpersons on their satellite practices found diverse approaches: 50% of chairs hired physicians to practice only in satellites, 50% of satellites did not have resident or fellow trainees, and 80% of physicians at satellites were on a clinical track.^1,7^ While these findings provided a glimpse into the views and goals of ophthalmology chairs, input and perception of satellite physicians were missing. Publications have described the importance of aligning priorities and values in academic medicine to enhancing faculty satisfaction and retention and to improving the health of academic medical centers as well.^14–18^

This pilot study aimed to explore the perceptions of ophthalmologists at satellite offices of a large academic department regarding compensation, satisfaction with work at satellite offices, academic and administrative departmental support, patient wait times and satisfaction, and their understanding of operations management.

## Methods

This pilot study of satellite clinics of the Department of Ophthalmology and Visual Sciences at the University of Michigan was based on a questionnaire sent to all ophthalmologists in the department who work least 1 day per week at a satellite location. There are 10 satellite offices, and each satellite office is staffed by 1 to 10 doctors per week. Each satellite is led by a medical director, an ophthalmologist working there the majority of his/her work week. One satellite has an ambulatory surgery center (ASC). Five satellites are stand-alone ophthalmology clinics; five satellites are in multispecialty buildings.

All satellites are within 30 miles of the main campus in Ann Arbor, MI, USA, except for one more than 50 miles from campus. Satellite offices in this department provide a variety of ophthalmology subspecialties in addition to comprehensive eye care, which is the most common offering. Seven of 10 satellites offer cornea, glaucoma, and retina subspecialties. Some satellites offer oculoplastics, optometry, pediatric ophthalmology, and low vision services. Faculty in this department are categorized as clinical (nontenure track) or academic (tenure track).

The questionnaire was validated and consisted of 44 questions (Table 1); select questions are included. Physicians were asked to answer the questions to the best of their ability; space for free-text answers was provided. Some questions asked for a response on a 5-point scale, with 5 being the highest quality or most satisfaction and 1 being the lowest. The survey was sent electronically between September 1, 2019, and November 1, 2019. The survey was sent in a method that preserved confidentiality and prevented the authors from contacting individual respondents.

**Table 1.** Questions from Survey of Satellite Faculty Members in an Academic Ophthalmology Department

Question
1. What is your academic title? (faculty, affiliated, other)
2. Are you on a clinical or instructional track?
3. How many satellites do you currently staff? (1, 2, 3, 4, 5, 6, 7, 8)
4. On average, how many days per week do you work at the satellite location? (1, 2, 3, 4, 5, 6, 7)
5. When you do work at the satellite ophthalmology clinic, which of these best approximates your working hours? (full day, half a day, afternoon/evening only)
6. How far is the satellite from the institution's main location? (5–10, 10–20, 20–40, 40–60, >60 miles)
7. For each day of the week, which option most closely matches the hours of operation of the satellite ophthalmology clinic? (8:00 am–5:00 pm, 5:00 pm–7:00 pm, 7:00 pm–9:00 pm)
8. Is the building leased or owned?
9. How was the satellite acquired? (acquired private practice, new building, other)
10. What do you believe were the reasons for acquiring or building the satellite? (financial boost, volume, community outreach, campus expansion, other/free text)
11. Do you feel there is room for expansion of the satellite? (Yes/No)
12. At what facilities do ophthalmologists at the satellite operate? (main hospital, local hospital, ASC)
13. How does the ophthalmology department market the satellite in the community? (newspaper, television, internet, word of mouth, other/free text)
14. Are there clinical trials or research occurring at the satellite? (Yes/No)
15. Are there teaching opportunities at the satellite? (ophthalmology residents, medical students, internal medicine/family medicine residents)
16. Which ophthalmology subspecialties do the satellites possess? (retina, cornea, glaucoma, pediatrics, low vision, oculoplastics, comprehensive, optometry, optical shop)
17. Do medical/surgical specialties other than ophthalmology share the building? (Yes/No)
18. How many patients would you say ophthalmologists at your satellite see on average in a given day? (5–10, 10–15, 15–20, 20–30, 30–40, 40–50, 50–60, 60–70, 70+)
19. Is there a “doctor of the day” model at the satellite location? (Yes/No)
20. Is after-hours ophthalmology call taken at the satellite? (Yes/No)
21. What support staff does the satellite ophthalmology clinic employ? (technicians, front desk, clinic manager, scribes, medical assistants)
22. How many staff members does the satellite ophthalmology clinic employ on a typical day you are working there? (number 0 to 10 for technicians, front desk, scribes, medical assistants)
23. How many billers are dedicated to the satellite? (1, 2, 3, 4, 5, 6)
24. Is billing centralized or local?
25. Are the following staff working part-time or full-time? (physicians, technicians, front desk staff, scribes, medical assistants)
26. Who runs the ambulatory care unit? (chairman, medical director, other/free text)
27. Where does the medical director live in proximity to satellite? (1–10, 10–20, 20–30, 30–40, 40–50, >50 miles)
28. What percentage of the ophthalmology department's revenue is generated from your ophthalmology satellite clinic? (<10, <25, 25–50, 50–75, other)
29. Are the following technologies used in the satellite and the main location the same? (EMR, phone system, testing/photography)
30. Would you say it is difficult to implement new technology at the satellite? (*e.g.*, EMR, new equipment, phone system, imaging system)
31. Where do the majority of referrals to the satellite practice come from? (internal, external, other/free text)
32. Where do the majority of your clinic referrals come from? (optometry, internal/family medicine, ophthalmology, other patients, other/free text)
33. How do you build relationships with referring doctors? (CME courses, meet and greet, dinners, meetings, referral letters, other/free text)
34. How satisfied are you overall with working at the satellite? (scale of 1–5 with 5 being the highest).
35. How satisfied are you with the satellite location geographically? (scale of 1–5 with 5 being highest)
36. How satisfied are you with the satellite facilities and equipment? (scale of 1–5 with 5 being highest)
37. How satisfied are you with the quality, capabilities, and training of staff at the satellite? (scale of 1–5 with 5 being highest)
38. How satisfied are you with your overall earning potential? (scale of 1–5 with 5 being highest)
39. Do you feel your salary is comparable with physicians who work exclusively at the institution's main location? (yes, no, other/free text)
40. How do salaries of the following staff compare between the satellite and main location? (technicians, medical assistants, scribes, front desk staff)
41. Rate your impression of patient satisfaction at the satellite versus main location (1–10, 10 being best)
42. Average wait time for new patient appointment at the satellite versus main location (1–3 days, 1 week, 2–3 weeks, 1 month, 2–3 months, 4–6 months)
43. Average wait time from check-in to doctor visit at satellite versus main location (10–20 minutes, 20–30 minutes, 30–40 minutes, 40–60 minutes, 1–1.5 hours, 1.5–2 hours, 2–3 hours, 3–4 hours, 4–5 hours)
44. How do you stay connected with the university? (research, meetings, social gatherings, surgery, other/free text)

ASC, ambulatory surgery center; CME, continuing medical education; EMR, electronic medical records.

The Institutional Review Boards of the University of Michigan Medical School determined this work qualified as exempt research because the survey posed little to no risk to respondents. The eventual goal was to expand the survey to satellite ophthalmologists in other academic departments.

## Results

The survey was sent to 32 eligible physicians who worked part-time between satellites and the main campus or full-time at satellites. Seventeen (53%) of the satellite ophthalmologists were female compared with less than half of the overall department. Seventeen (53%) physicians completed the survey; the response rate for each question ranged from 29% (question regarding how many days a week are spent at a satellite) to 100%, with a median response rate to questions of 94%.

### Satellite physician characteristics, job satisfaction, and perceptions of satellite efficiency, staff, compensation, and connection with department

The majority (81%) of respondents were on the clinical track and worked full days rather than half-days while at a satellite. A little more than half of respondents worked 1 day a week at a satellite, while the rest worked between 3 or 5 days at a satellite. More than half of the ophthalmologists responded that they saw 20–30 patients per day; however, one ophthalmologist reported consistently seeing 70+ patients per day. Some respondents felt that they tolerated a long commute to work because the satellites were run in a very efficient manner, allowing them to see more patients per day.

A few satellite ophthalmologists conducted an evening weekday clinic ending at 7 p.m. One ophthalmologist, however, stated that it was more challenging to maintain a steady volume of patients at his/her satellite location compared with practicing at the main campus. Two ophthalmologists felt that compared with work at an ophthalmology-only satellite, work at a satellite in a multispecialty building was more difficult because of less autonomy and because of shared personnel such as front desk and call center staff that were not specific to ophthalmology.

Overall satisfaction of ophthalmologists working at the satellites was 3.6 out of 5. Ratings of the satellite facilities, equipment, and quality and capabilities of the staff were 3.9 out of 5. More than 60% of respondents felt that implementing a new technology at satellites was not difficult. More than 90% of satellite ophthalmologists believed that the staff at satellite clinics were well-trained and that the salaries of the front desk and technicians were comparable between satellites and the main campus.

The lowest rating (3.2 out of 5) was regarding satisfaction of overall earning potential at the satellites. The majority (71%) of respondents felt their own earning potential was comparable with what they could earn at the main campus. A few satellite ophthalmologists relayed concerns about salary, volume, and geographic location. This minority felt their total compensation was low compared with doctors at the main campus. They cited the low-volume potential at certain satellites from local market saturation and competition with surrounding private practices as reasons for not meeting target relative value units, and hence, not earning their incentive pay, which is based on productivity. Fewer than 10% of satellite ophthalmologists responded that research was being conducted at satellite clinics.

While satellite doctors taught internal medicine, family medicine residents, or medical students in their clinics, satellite doctors acutely felt the lack of interaction with ophthalmology residents. The majority said they felt connected to the main campus through performing surgery or attending department meetings there in person. More than three-fourth of the faculty who completed the survey selected “meetings” as the only way to stay connected with the rest of the ophthalmology department.

### Satellite physician understanding of operations management and perceptions of patient referrals, satisfaction, and access

Satellite physician perceptions regarding rationale for ophthalmology satellite office acquisition or development varied widely. The majority of satellite physicians believed the goal of satellite acquisition or development was to boost the department's patient volume and to expand the department's physical footprint. Many respondents believed that departmental financial gain or community outreach was a secondary reason for establishing satellite practices.

About 10% of respondents did not know whether their satellite was leased or owned. More than 50% of respondents could not estimate the percentage of department revenue that satellites generated. Those who responded believed that satellites generated under 25% of the department's total revenue. In addition, the majority did not know whether billing was done locally or at the main campus.

Respondents said the majority of referrals were internal; the next highest referral sources were internal medicine and family medicine. Three-quarters of satellite ophthalmologists said they built relationships with doctors through referral letters they generated themselves. Others lectured at local continuing medical education courses or participated in “meet and greets”—short, informal events for satellite ophthalmologists to meet with community doctors. About three-quarters of respondents felt their new patients came to them through “word of mouth,” for example, other patients. Internet, newspaper, and television advertisements were considered less common means of generating patients.

One-third of respondents believed the wait time for their patients to be seen once they checked into their satellite office was 10–30 minutes versus 1–2 hours at the main campus. More than half of the respondents rated patient satisfaction at a level of 8/10 at satellites, while the rest gave a satisfaction score of 9/10. Satellite ophthalmologist perception of patient satisfaction at the main campus ranged from 5/10 to 9/10, with the majority rating it as 8/10.

## Discussion

This pilot study queried ophthalmologists working at the satellite practices of an academic ophthalmology department to investigate possible factors key to the success of such clinics, which are becoming an integral part of many academic ophthalmology departments, and to the success of the physicians who work there. Because of the effort and expense to recruit physicians and concerns about physician burnout,^19^ physician satisfaction and perceptions about their practice setting are as important as metrics of efficiency and patient access to care. The majority of satellite physicians in this department reported being satisfied with work at satellite offices and felt they were providing high-quality care in a more efficient manner compared with the main campus.

Some satellite ophthalmologists, however, expressed concerns about salary, volume, and geographic location because of competition from private practice groups nearby. There was also misunderstanding among satellite doctors regarding expenses, medical billing and production, staff compensation, revenue contribution of satellites to the department of ophthalmology, and their own compensation. As no residents or fellows go to satellite offices, lack of involvement in teaching and research missions was another concern especially as academic medicine has traditionally had a tripartite mission. Mentorship and programming regarding operations management, marketing, and business practice should be tailored to this group of faculty with a goal toward retention and satisfaction. Although responses were anonymized, the majority of satellite doctors are clinical, female, and junior in rank compared with doctors at the main campus. Women in business and academic settings lag behind men in access to quality mentorship and effective networks.^20,21^

Traditionally, professional development for academic physicians has most often occurred through mentoring and networking. Therefore, the demographic features of satellite physicians are important considerations; by definition they are not at the main campus where traditional mentoring and networking occur.

Patients are inclined to seek care at satellites for many reasons: shorter driving distance, free and ample parking, access to quality of care similar to that offered at the main campus, and timeliness of appointment and examination.^3,5^ The perception of most ophthalmologist respondents was correct: patient wait times between check-in and physician examination are better at satellite locations than at the main campus. By examining electronic time stamps at ophthalmology-only satellites, we discovered that patient check-in to check-out was on average 20 minutes faster than at the main campus.

Five satellites were located in multispecialty office space, which would not have provided comparable information. The average length of time for a new ophthalmology patient appointment was under 1 week at a satellite location compared with 2–3 weeks at the main campus. Whereas one clinic conducted a weekday evening clinic, there were no after-hour ophthalmology clinics at the main campus. One academic pediatric radiology division reported increased referring physician and patient satisfaction with satellite development in the community,^22^ which is likely true for this ophthalmology department as well. As medicine is adapting to an era of patient-oriented outcomes and emphasis on patient satisfaction,^23^ wait time and access to care are important metrics, and patient experiences at satellite offices contribute to perceptions of the overall department.

Most physicians in this ophthalmology department were satisfied with their work at a satellite office. The majority believed that nonphysician employees at satellite offices performed at a higher level than employees at the main campus. A small number believed that satellite staff were paid less than at the main campus, which is not true; salaries are based on seniority, regardless of location. A minority of respondents were unaware whether satellites were owned or leased; 9 of 10 satellites operate in buildings owned by the university. In addition, most respondents did not know whether billing was performed centrally, not locally (the answer is that satellite billing is done centrally). About half of the satellite doctors were unaware of the revenue contribution of satellites to the overall department, which at the time of this writing is about 25%.

These responses suggest that satellite doctors need to be empowered with more financial tools and operations management skills to help them practice at their satellite. Satellite doctors tend to be more junior than the rest of the department,^1^ which may limit their level of sophistication about these matters unless department administrators expend effort. Being armed with this type of knowledge and skill may help satellite doctors feel more vested in their work.

The majority of respondents felt that their compensation (base salary plus a supplement based on productivity—“bonus”) was comparable with that of ophthalmologists at the main campus. A few satellite ophthalmologists expressed concerns about their earning potential because of their location in highly saturated markets and felt that their total compensation was low compared with colleagues at the main campus. This concern may be associated with the perception of some satellite doctors of having little marketing support. Marketing and administrative support tailored to individual locations likely would particularly benefit those practicing in direct competition with busy private practices. However, other factors may hinder productivity and total compensation, such as physician burnout, physician preference regarding clinic volume, and academic and personal commitments. Analysis of these factors was beyond the scope of this study.

Another perception of these satellite ophthalmologists was that compensation increases with a higher academic rank, which is not entirely accurate. Academic rank determines base salary to the extent that the base salary of physicians who stagnate in academic rank also stagnates. More important than increasing one's base salary, however, is that advancement provides leadership opportunities inside and outside the department, and publications and grants are linked to promotion to upper ranks and to appointment to leadership positions.^24^ In studying faculty members in leadership positions in 16 divisions of a large department of medicine, authors found that being mired in often time-consuming activities attached to lower level leadership positions could deter from productivity in terms of publications and grant awards.^25^ Leadership roles offer visibility and very likely affirm faculty members' sense of being valued.

Despite concerns, compensation is not a reason for joining an academic department; satellite faculty, for example, could easily work in private practice if compensation were their priority. Although satellite clinics offer the exciting possibility of expanding patient participation in clinical research, the perception of satellite ophthalmologists in this department was that little research was conducted by satellite doctors. Involvement in research, however, is important because promotion is tied to scholarly activity. Comparing male and female faculty in academic medicine, women were “more likely to be retained and to achieve senior rank if they are more academically productive, regardless of whether they pursue research, education, or clinical academic pursuits.”^26^ As noted in a survey of ophthalmology chairpersons, however, research endeavors and trainee education are not considered to be priorities of satellite practices.^7^

In the future, participation in clinical research may become more common at these satellite clinics. However, because most satellite doctors in this department and others are junior in rank, it is critical for them to have mentorship, an important factor in academic success and faculty satisfaction in academic settings, regardless of gender.^27^ If satellite faculty are held to the same academic standards—to contribute substantively to academia's tripartite mission—then they need opportunities and support to advance toward promotion.^7,13^ In addition, satellite faculty must be thoroughly integrated into the departmental culture so that they remain engaged and feel valued.^10–13,28^ Satellite faculty surveyed said they try to stay “connected” with the main campus, but they may need to expend extra effort to find mentors and perhaps the department can promote mentorship of this set of faculty.

A recent article on female faculty at a large academic center did not delve into details of clinicians or satellite offices, but it did uncover gender differences in the domains of leadership, promotion, and faculty satisfaction. Recommendations included mentorship, sponsorship, clear leadership charts, and an effort to make salary/compensation plans, resource allocation, and administrative support transparent because female faculty reported dissatisfaction with regard to resource allocation, leadership, participation in decision-making, and mentoring.^29^ Changes of this nature would benefit all faculty, not just female faculty. Both compensation models and pathways for academic advancement should be transparent to faculty as both are major factors in faculty physician satisfaction,^30^ which in turn affects retention. Anecdotally, satellite faculty turnover is already noted to be higher than at the main campus for many ophthalmology departments.

Faculty development needs to be tailored to the characteristics of satellite ophthalmologists, which include being clinicians of junior rank (many having just finished residency or fellowship) and majority female. Institutions may contribute to the success of women leaders by addressing four key areas that include (1) “equipping” the women with development opportunities, training in negotiation, strategic career planning in early stages of an academic career to internal and external national programs such as the Executive Leadership in Academic Medicine (ELAM) and the Association of American Medical Colleges Women in Medicine program in later stages, (2) creating equal opportunities, (3) valuing relational skills and increasing visibility, and (4) assessing and revising work culture.^31^

All of these areas of focus are critical to faculty who are off-campus, often junior in rank, and mired in clinical work. Twenty-nine of the 32 faculty members at satellite clinics were clinical assistant or clinical associate professors, which is a nontenured track. Only three were on the academic track and were assistant and associate professors. So that faculty members could provide candid responses without fear of retribution or retaliation, the invitation to participate promised anonymity. Because of small numbers (32 total satellite faculty members), we did not ask for gender or academic rank as they could be identifiers, and therefore, we could not examine for any correlation of responses with gender or rank. It is possible a larger study of many departments' satellite practices (through which participant numbers approximate several hundreds) might obviate such concerns of identification.

One limitation of this survey is its small sample size. Results of this pilot study, however, will serve as the basis of a future study to query satellite ophthalmologists in other academic departments. The response rate of 53% in this survey is comparable with that published of surveys of deans and chairs.^7,32–34^ Another limitation is the paucity of questions regarding teaching opportunities at satellites. According to a 2015 survey of ophthalmology chairpersons, residents and fellows were sent to satellites in 50% of departments with satellite offices. Satellite offices offer a unique opportunity for trainees to work in settings with increased efficiency although some chairmen noted that their presence “would decrease the [satellite] practice's efficiency.”^7^ A balance likely can be struck for satellites to be both a model for efficiency and a setting for trainee education.

## Conclusion

Satellite offices are an integral part of many academic ophthalmology departments in the United States and may offer efficiencies and access that clinics at the main hospital cannot offer.^35^ Academic departments are able to increase access to care when they build satellites in communities, which is important as the population grows outside urban centers and because the main campus is often unable to expand in older neighborhoods. In addition, patient satisfaction may be higher at satellite clinics than the main campus because of access and wait times. Moreover, in the event of a pandemic such as COVID-19, satellite clinics may allow earlier return to clinical activity compared with the main campus; continuity of important clinical services; and preservation of revenues for the department and university.^35^

The doctors in satellite offices of many (if not all) academic departments of ophthalmology are self-supported through clinical work even as faculty members. This is not true of faculty at the main campus. Satellite doctors in some departments of ophthalmology additionally pay for clinic space and staff on top of their own salary support. Departments should examine ways to keep satellite physicians vested despite physical separation from the main campus and pay special attention to the demographics of this group of faculty—junior, clinical, and female.^7^ The opportunity for satellite ophthalmologists to spearhead community-based clinical research supposedly exists and would help fulfill the tripartite mission of academic medicine. However, mentorship is necessary for this endeavor. While most satellite physicians in this department were satisfied with their work and felt confident they were working with excellent staff to provide efficient care with higher patient satisfaction than at the main campus, some satellite physicians expressed concerns about compensation and marketing support especially in locations with highly competitive private practices.

“Financial success, the measure of the marketplace, has become the dominant standard of measurement or ‘value’ for most academic medical centers and physicians,” wrote Souba 20 years ago.^17^ If so, survey responses indicated that some satellite doctors of academic departments lack information on operations management or the financial status of their satellite, which, unlike divisions at the main campus, is key to the survival of a satellite.^7^ In addition to providing resources to support the academic careers of satellite faculty members, department leaders and administrators should provide adequate staffing and marketing to maintain efficiency and patient access, two domains that make satellite offices attractive to patients and valuable to academic departments.

## Abbreviations Used

ASC: ambulatory surgery center
CME: continuing medical education
EMR: electronic medical records
IM: internal medicine
MA: medical assistant
